# Multi-Level Transformer-Based Social Relation Recognition

**DOI:** 10.3390/s22155749

**Published:** 2022-08-01

**Authors:** Yuchen Wang, Linbo Qing, Zhengyong Wang, Yongqiang Cheng, Yonghong Peng

**Affiliations:** 1 College of Electronics and Information Engineering, Sichuan University, Chengdu 610065, China; wangyuchen98@stu.scu.edu.cn (Y.W.); wangzheny@scu.edu.cn (Z.W.); 2 Department of Computer Science and Technology, University of Hull, Hull HU6 7RX, UK; y.cheng@hull.ac.uk; 3 Department of Computing and Mathematics, Manchester Metropolitan University, Manchester M1 5GD, UK; y.peng@mmu.ac.uk

**Keywords:** social relation recognition, data driven, social intelligence, transformer

## Abstract

Social relationships refer to the connections that exist between people and indicate how people interact in society. The effective recognition of social relationships is conducive to further understanding human behavioral patterns and thus can be vital for more complex social intelligent systems, such as interactive robots and health self-management systems. The existing works about social relation recognition (SRR) focus on extracting features on different scales but lack a comprehensive mechanism to orchestrate various features which show different degrees of importance. In this paper, we propose a new SRR framework, namely Multi-level Transformer-Based Social Relation Recognition (MT-SRR), for better orchestrating features on different scales. Specifically, a vision transformer (ViT) is firstly employed as a feature extraction module for its advantage in exploiting global features. An intra-relation transformer (Intra-TRM) is then introduced to dynamically fuse the extracted features to generate more rational social relation representations. Next, an inter-relation transformer (Inter-TRM) is adopted to further enhance the social relation representations by attentionally utilizing the logical constraints among relationships. In addition, a new margin related to inter-class similarity and a sample number are added to alleviate the challenges of a data imbalance. Extensive experiments demonstrate that MT-SRR can better fuse features on different scales as well as ameliorate the bad effect caused by a data imbalance. The results on the benchmark datasets show that our proposed model outperforms the state-of-the-art methods with significant improvement.

## 1. Introduction

A social relationship, as a key concept in sociology, describes the interaction between people. It has been proved to have both short-term and long-term effects on human health [1]. Understanding the social relationships among people is thus essential for identifying the link between social relationships and health outcomes. In addition, effective social relation recognition (SRR) can also provide valuable interactive information for other related tasks, such as an activity analysis [2] and group emotion detection [3], which further benefits more comprehensive tasks, such as smart city design [4] and social sustainability [5].

Meanwhile, with the development of the Internet and multimedia, various platforms, e.g., Facebook, Twitter and TikTok, are generating huge amounts of social data with great application values [6]. Specifically, the different types of social data, including social network information (positioning information [7,8] and network graph structure [9]), text [10,11], image [12] and video [13,14], contain abundant interactive information between users and are conducive to understanding social relationships. Among these different forms of data, visual data reflect the relationship between individuals more intuitively than textual and social network information. Furthermore, compared with video, images show less complexity and are easier to be processed. In other words, recognizing social relationships based on images balances the intuitiveness and the complexity.

Existing methods for SRR based on images have their own paradigm, which contains three key parts: **(1) feature extraction, (2) feature fusion and (3) classification and optimization**. In terms of the scale of features, different features can be divided into intra-relation features, inter-relation features and scene features. A detailed classification of these features will be given in the related work.

**Figure 1:**
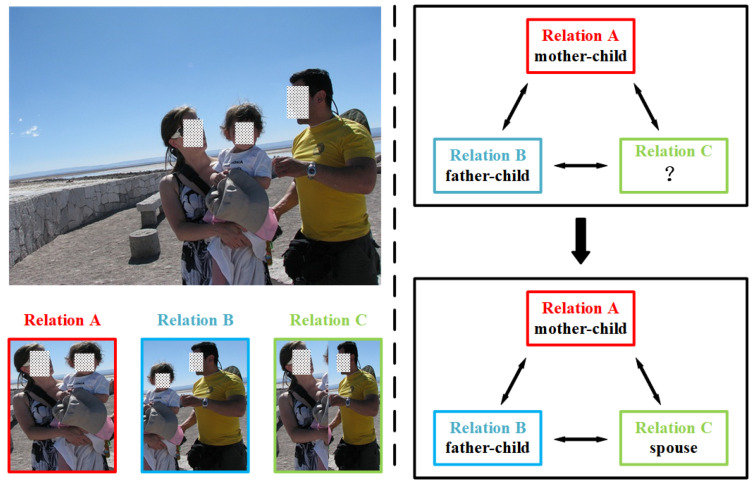
An example of the logical constraints among social relationships in one image. With relation A and relation B (‘*mother-child*’ and ‘*father-child*’ in the image), we can easily infer that relation C belongs to ‘*spouse*’ by the logical constraints. Note that all the displayed pictures are picked from People in Social Context (PISC) dataset.

For feature fusion, early attempts concatenate intra-relation features and scene features [15] or design simple feature selection methods to fuse them [12,16]. Recent works further exploit the inter-relation features (logical constraints, illustrated in Figure 1) by concatenating intra-relation features to generate relation nodes and introducing a gated graph neural network (GGNN) or a graph convolutional network (GCN) to propagate the messages among these nodes or to extract the topological information [17,18,19]. However, the above methods cannot effectively fuse the intra-relation feature to better represent social relationships because they ignore the varying degrees of importance of different features to a particular relationship. In addition, the updating mechanism of a GGNN and GCN inadequately considers the different influences of all the other neighbor nodes, namely the message-passing method among nodes is unreasonable when exploiting logical constraints for SRR.

For classification and optimization, using standard cross-entropy (CE) to train the multi-layer perceptron (MLP) or fully-connected layer (FC) is the mainstream [12,16,17,18,19]. However, the benchmark datasets for SRR have imbalanced sample numbers across different classes, which means the dominant classes will overwhelm the training process and thus cause low accuracy of classes with fewer samples. In addition, samples from different specific classes have similar visual clues, e.g., samples from the class ‘*friend*’ and samples from the class ‘*couple*’. High inter-class similarity leads to serious confusion between these similar classes, which can be found in the confusion matrixes in [17,18]. The above methods show the absence of consideration for the bad effect caused by imbalanced data and high inter-class similarity.

In this paper, we propose a Multi-level Transformer-Based Social Relation Recognition model (MT-SRR), which introduces a transformer into the feature extraction module and feature fusion module in different ways, as well as design a new loss function for relation classification. Specifically, the vision transformer (ViT) [20] is adopted to globally extract the visual features of persons. An intra-relation transformer (Intra-TRM) is then introduced to fuse intra-relation features and scene features to generate more rational social relationship representations. Then, an inter-relation transformer (Inter-TRM) is designed to enhance inter-relation features by attentionally aggregating similar social relationship representations in the same image, which has logical constraints among them. Finally, margins related to sample similarity and sample numbers are added to the standard CE in order to adaptively increase the distance between different classes with consideration of the imbalanced data.

Our contributions can be summarized as follows:

- A new transformer-based feature fusion block (Intra-TRM) is proposed to carefully fuse the intra-relation features and scene features in order to generate better social relation representation. The designed module dynamically fuses these extracted features, which give different features weights related to their similarity to the key features of a specific relationship.
- A new transformer-based inter-relation feature enhancement block (Inter-TRM) is employed to enhance the representation of similar relationships in one image and exploit the logical constraints among them. This module attentionally aggregates similar relation representations in the same image, which can solve the problem caused by the unweighted updating mechanism of a commonly used graph-reasoning network for SRR.
- A new margin is designed to mitigate the negative effect caused by imbalanced data. The new margin is related with inter-class similarity and influenced by the sample numbers, which can adaptively adjust the distance between different classes with different sample numbers.
- Our proposed MT-SRR achieves the state-of-the-art results on two public benchmark datasets for SRR, i.e., the People in Social Context (PISC) [12] and the People in Photo Album (PIPA) [21]. Extensive ablation results further demonstrate the effectiveness of the Intra-TRM, Inter-TRM and the newly designed loss function.

The rest of the paper is organized as follows. Section 2 reviews the related work about SRR and the applications of a transformer in computer vision. Section 3 elaborates the details of our proposed MT-SRR. The detailed experimental results are described in Section 4. Section 5 gives the conclusion of this paper.

## 2. Related Work

In this section, we give a holistic view of social relation recognition to describe the tendency of its development, followed by a literature review of a transformer used in computer vision, which can be introduced to better orchestrate the intra-relation features, inter-relation features and scene features for SRR.

### 2.1. Social Relationship Recognition

Social relationship recognition is now a field of growing interest to the research community. In this subsection, we will brief the SRR in terms of the three key parts of the paradigm, as mentioned in Section 1.

Through years of researchers’ persistent efforts, the specific categories of features have been richly extended, as shown in Table 1. In detail, earlier attempts tend to manually design face features, e.g., the colors of skin and hair [22] and appearance [23], to recognize simple kinship relationships. With the increasing demand for detailed relation recognition and the development of a deep learning network, researchers began to use complex neural networks to extract face features for detailed relation recognition. Gao et al. [24] introduced a higher-order graph neural network to find the connection between two faces. After the publications of the PISC datasets [12] and the PIPA datasets [21], researchers began to pay more attention to extracting whole body features and scene features. Li et al. [12] adopted a convolutional neural network (CNN) to extract body features from cropped individual regions and union regions as well as extract visual scene clues from cropped contextual objects. Zhang et al. [25] further utilized the pose key points to enhance the body features and extract scene information from the whole image. Goel [15] recognized age and gender clues and extended SRR to a multi-task framework. Since then, the performance of intra-relation features extraction was close to a saturation point and subsequent works started to take inter-relation features into consideration. Li et al. [19], Qing et al. [17] and Li et al. [18] successively constructed different graph structures to generate the logical constraints among different types of social relationships.

For feature fusion, most works focus on the fusion of concatenated intra-relation features and scene features. Li et al. [12] adopted the traditional attention mechanism to fuse the concatenated intra-relation features and contextual object clues. Wang et al. [29] introduced a gated graph neural network (GGNN) to pass messages between intra-relation features and contextual objects. Few methods try to better fuse the intra-relation features but neglect the fusion of inter-relation features, e.g., Wang et al. [16] learned a sparse weighting matrix to select optimal feature subsets in order to reduce the noises and redundancy caused by high-dimension multi-source attributes. Recent methods employ different variants of a graph neural network (GNN) to grasp the inter-relation features and fuse them (provided within the GNN itself), e.g., Li et al. [18] designed a new weighted-GGNN to attentionally fuse inter-relation features and scene features. Qing et al. [17] simultaneously utilized a GGNN and graph convolutional network (GCN) to fuse the global and local information among inter-relation features.

The aforementioned SRR methods have validated the effectiveness of features on different scales and have achieved some progress on the fusion of concatenated intra-relation features, inter-relation features and scene features. However, few works take the effective fusion of intra-relation into account. Moreover, the updating mechanism of existing social relationship graph-reasoning methods [17,18,19] still inadequately considers the different influences of all the other neighbor nodes, although Li et al. [18] have introduced different weights between the scene node and relation nodes. Furthermore, existing works rarely attempt to alleviate the problem caused by imbalanced data and high inter-class similarity.

### 2.2. Transformer for Visual Tasks

Significant success has been achieved by the transformer in computer vision led by the ViT. Firstly, various transformer-based backbones greatly improve the performance of feature extraction. The great improvement is credited to multi-head self-attention (MSA) because this structure can simultaneously calculate self-attention among all the patches and thus fuse the global feature of the whole images. Subsequent methods integrate the design philosophy of a CNN into a transformer structure and a series of variations [30,31,32] of ViT have been proposed as the backbones for feature extraction.

Secondly, the transformer structure also benefits a large number of downstream tasks, e.g., semantic segmentation [33], remote sensing image classification [34,35,36] and behavior analysis [37,38,39]. However, in tasks such as semantic segmentation and remote sensing image classification, the contribution of a transformer structure is still limited to its advantage in visual features extraction. On the contrary, in behavior analysis, due to the similarity between video frames and image patches (both are parts of the whole video stream or image), the transformer structure is introduced to exploit the temporal information among these video frames [38]. Similarly, a transformer is also employed to exploit the features from the pose skeleton in order to recognize human actions [39].

The above applications of the transformer structure have proved its potential capacities for feature extraction and feature interpretation. In terms of SRR, using a transformer-based backbone can exploit more global information hidden in images compared with CNN-based backbones, which contain the important interactive information between individuals. MSA, as the core of the transformer structure, also enables the transformer to attentionally fuse intra-relation features and inter-relation features, when the input is various features and relation representations, respectively. To this end, we first introduce the ViT as the feature extraction module. Intra-TRM is then employed to attentionally fuse intra-features with the ability of MSA. Finally, Inter-TRM is designed to enhance the representation of a similar relationship in one image for more rational social relation recognition.

## 3. Methods

In this section, we elaborate on the proposed MT-SRR. We give a general view of the whole framework with a brief introduction of the design process, followed by a detailed description of three key parts in our model, namely (1) feature extraction, (2) feature fusion and (3) classification and optimization.

### 3.1. Overall Framework of Model

**Figure 2:**
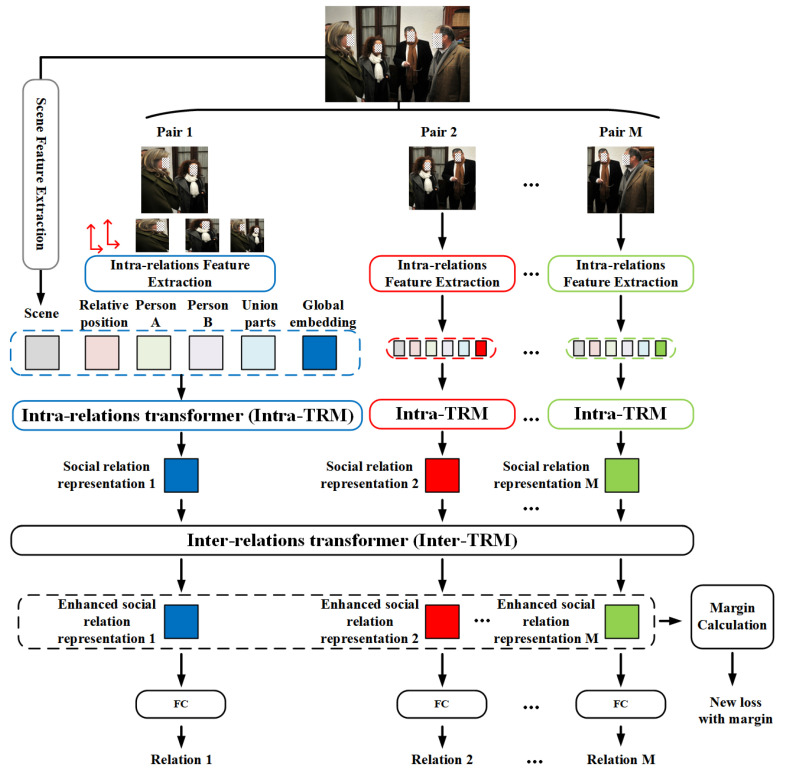
Overall Framework of MT-SRR. Blocks with solid lines are real computation modules, while blocks with dash lines mean the contents are utilized together. For each person pair in the image: (1) We first extract four intra-relation features and one scene feature. (2) Along with a learnable global embedding, all the extracted features are fed to Intra-TRM, which outputs the learnable global embedding as fused social relation representation. (3) All the social relation representations in the same image are fed to Inter-TRM to further enhance the social relation representation. (4) Finally, the output of Inter-TRM, i.e., enhanced social relation representations, is classified into specific relations. Specially, we utilize the outputs of Inter-TRM and the sample number to redesign a new loss with an adaptive margin. The figure is best seen in color.

Similar to the general paradigm for SRR [12,17,18,19,25], the proposed MT-SRR pays more attention to recognizing pair-wise relationships, whose overall framework is depicted in Figure 2. Specially, we adopt two transformer-based feature fusion models on two levels: one is used to fuse the intra-relation features and scene features, and the other is utilized to fuse the inter-relation features to enhance the representation of a similar relationship in one image. Briefly speaking, for an image with *N* individuals, there are $M=C_{N}^{2}$ different relationships (‘*no relation*’ is treated as a special kind of relationship in this paper). For each social relationship, we first adopt pretrained ViTs to extract different intra-relation features for its capacity of globally exploiting the visual clues and employ a ResNet50 pretrained on Places365-Standard [40] especially for scene recognition. Then, Intra-TRM is used to attentionally fuse the output of the feature extraction module, namely the intra-relation features and scene features, and generate a well-designed relation representation. Next, Inter-TRM is employed to enhance the relation representations with inter-relation features by attentionally fusing similar relationship in the same image and generating a new relation representation. Finally, the outputs of Inter-TRM are fed to the classification module. At the same time, we accumulate the sample numbers of different relationships and calculate the average cosine similarity among the outputs of Inter-TRM. A dynamic margin related to the sample numbers and average cosine similarity is then added to standard CE in order to alleviate the bad effect caused by data imbalance.

### 3.2. Feature Extraction

**Figure 3:**
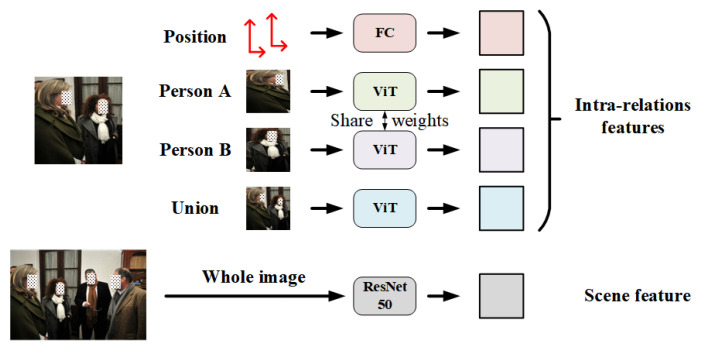
Illustration of feature extraction. Note that here we only take one person pair in the input image as an example.

For a specific relationship in an image with *N* individuals, we extract four different intra-relation features and one scene feature by five channels, as sketched in Figure 3. Specially, we first crop the image with the bounding boxes information provided by the labels and generate two individual regions and a union region of two individuals. Individual regions contain the visual clues of a single person, e.g., face, clothing and pose, while the union region implies the interactive information between two individuals. These cropped regions, along with the whole images for scene feature extraction, are uniformly resized to 224×224 as the input of specific feature extraction networks. Relative position information, including the coordinates and areas of two individual bounding boxes, are also fed to the feature extraction module.

Different from recent SRR methods [17,18], we introduce fine-tuned ViT pretrained on ImageNet [41] to extract intra-relation features. Compared with CNN, ViT divides the image into small patches and employs multi-head self-attention (MSA) to more globally integrate the features from different patches, which pays more attention to the global interactive information and thus benefits the social relation representation. In our framework, the output dimension of the last MLP layer in ViT is changed from 1000 to 2048 and the parameters of MLP layer are fine-tuned during the training process to adapt to our tasks. Scene feature is still extracted from the whole image by a ResNet50 pretrained on Place365-Standard dataset and we change the output dimension of ResNet50 to 2048 by removing the last classification layer and the first pooling layer. Here, we do not use the ViT as a scene feature extraction network because the scene information is relatively simple and Place365-Standard dataset is specially proposed for scene recognition, which provides pretrained models using ResNet50 as the backbone. In addition, an FC, whose output is a vector with the size of $R^{2048}$, is adopted to extract the relative position information. Finally, we obtain four $R^{2048}$ intra-relation features and one $R^{2048}$ scene feature for each relationship in the image, which are fed to subsequent feature fusion module.

### 3.3. Transformer-Based Feature Fusion

Next in the pipeline is the features fusion module. We first design a transformer-based feature fusion module, namely Intra-TRM, to dynamically fuse all the features fed by the feature extraction module and generate more rational social relation representations for each relationship in an image. Then, another transformer-based feature fusion module, i.e., Inter-TRM, is introduced to enhance the social relation representation generated by Intra-TRM, which utilizes MSA to attentionally aggregate similar social relation representation in the same image. The details of the whole module are elaborated as follows in terms of Intra-TRM and Inter-TRM.

For Intra-TRM, the inputs are the intra-relation features and scene features in previous steps. Inspired by the application of transformer structure in Natural Language Processing (NLP) [42], we add an extra global embedding $x_{global}$ with the same dimension as those extracted features to the input, for globally fusing all the extracted features for each relationship in one image. The whole input of Intra-TRM ($z_{input\_intra}$) can be expressed as:

(1) $$z_{input\_intra}=\left[x_{global\;};x_{1};x_{2};x_{3};x_{4};x_{scene\;}\right],\;x_{global\;},x_{1},x_{2},x_{3},x_{4},x_{scene\;}\in R^{M\times 2048}$$

where $x_{1}$, $x_{2}$, $x_{3}$, $x_{4}$, $x_{scene}$ are the features extracted from two individual regions, one union region, relative position and the whole image, respectively. *M* is the number of relationships in an image with *N* individuals, as mentioned in Section 3.1.

Then, we utilize a stacked transformer to globally fuse the intra-features and scene features for more rational social relationship representations. In addition, residual connections are added before and after every block, respectively. The whole process can be described by the following formula:

(2) $$z_{l}^{\prime }=\mathrm{MSA}\left(\mathrm{LN}\left(z_{l-1}\right)\right)+z_{l-1},\;l=1\cdots L$$

(3) $$z_{l}=\mathrm{MLP}\left(\mathrm{LN}\left(z_{l}^{\prime }\right)\right)+z_{l}^{\prime },\;l=1\cdots L$$

where *L* is the number of stacked blocks, which is set as 12, referring to [42]. $z_{l}$ denotes the outputs of the *l*-th block, while $z_{l-1}$ has similar meaning. MSA is extended by standard self-attention, which runs several self-attention operations (called ‘heads’) in different vector space in parallel and concatenates their output for subsequent processing. LN is the abbreviation of layer normalization.

**Figure 4:**
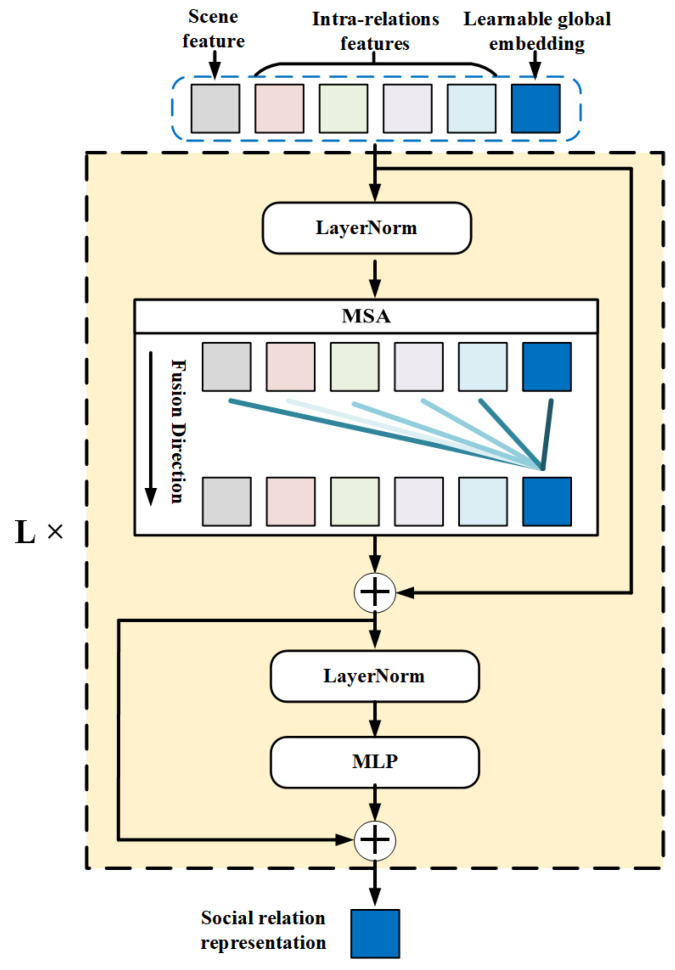
Intra-TRM module. For one person pair, the inputs are all the extracted features along with a learnable global embedding. With the MSA in stacked transformers, global embedding gradually fuses the various features with different weights.

Stacked transformer blocks ensure that the extra learnable global embedding can effectively fuse the intra-relation features and scene features with dynamic weights. For each relationship, we use the global embedding within the output of final transformer block as the social relation representation *r*. The illustration of whole Intra-TRM is shown in Figure 4.

For Inter-TRM, we use *M* social relation representations in one image as the inputs $z_{input\_inter}$, expressed as,

(4) $$z_{input\_inter}=\left[r_{1},r_{2},\cdots ,r_{M}\right],r_{i}\in R^{2048},i\in \left(1,2,\cdots ,M\right)$$

**Figure 5:**
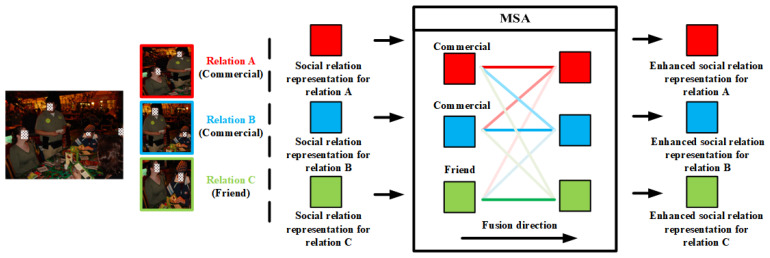
Inter-TRM module. Note that the stack transformer structures are intentionally omitted here for brevity and an intuitive understanding of the module.

Similar to Intra-TRM, a stacked transformer structure is constructed with Equations (2) and (3), which utilizes the MSA mechanism to enhance similar social relation representations in the same image. MSA mechanism enables these social relation representations to attentionally aggregate the similar representations and thus generate enhanced social relation representations, which benefits the inter-relation feature fusion for SRR. For example, as illustrated in the left part of Figure 5, there are three different relations in the image, namely two pairs of ‘*commercial*’ and one pair of ‘*friend*’. In MSA blocks of Intra-TRM, the input representations aggregate all the representations based on the similarity among them. To be specific, the similarity between one social relation representation and itself is most likely to be the largest, followed by the similarity between social relation representations of the same class, while the similarity between social relation representations of different classes is the lowest. The different similarity enables the block to attentionally aggregate the similar social relation representations, as the different gradations of colors in Figure 5. However, such a method will be affected by the problem of high inter-class similarity, which may increase the confusion between similar classes. To tackle the problem, we further design a new loss function, which is elaborated in the next section.

### 3.4. Classification and Optimization

The aforementioned Inter-TRM outputs are the final social relation representations $r_{final}$, which are used to calculate the per-class probability $p_{i}$ with a soft-max function, expressed as:

(5) $$SR_{i}=\left\{p_{1},p_{2},\cdots ,p_{m}\right\}=\mathrm{softmax}\left(\mathrm{FC}\left(r_{final\;}\right)\right)$$

where $p_{j}\left(j=1,2,\cdots ,m\right)$ is the probability of the *j*-th class. *m* denotes the number of classes in different SRR tasks (3, 6 and 16 for PISC-C, PISC-F and PIPA, respectively). $SR_{i}$ means the final classification results with the max probability of the *i*-th sample.

In order to further optimize our model to alleviate the bad effect caused by imbalanced data, we add an adaptive margin δ related to the sample numbers and the inter-class similarity to standard CE, inspired by [43]. The margin should satisfy the following two properties: (1) the more similar the two classes are, the larger it should be; (2) between two similar classes, the margin of the dominant class (class with more samples) should be smaller than that of the minority class in order to enlarge the suppression of minority class over dominant class. Therefore, for a sample of class *y*, the new loss function with margin is designed as follows,

(6) $$\mathrm{loss}=\log\left[1+\sum_{y^{\prime }\neq y}e^{z_{y^{\prime }}-z_{y}+\delta }\right]$$

where $z_{y^{\prime }}$ and $z_{y}$ are the output of class *y* and class $y^{\prime }$ after FC in Equation (5).

The adaptive margin δ can be calculated as follows,

(7) $$\delta =\frac{num_{\max}}{n_{y}}\cdot cosine\_similarity\left(y,y^{\prime }\right)$$

where $num_{max}$ is the maximum sample number of different classes in training data, $num_{y}$ is the sample number of class *y*. $cosine\_similarity(y,y^{\prime })$ means the average cosine similarity between samples in class *y* and samples in class $y^{\prime }$.

## 4. Experiments and Results

In this section, we first brief two public benchmark datasets for SRR, i.e., the PISC [12] and PIPA [21] datasets, followed by the implementation details. Then, we present the results of the comparison experiments with state-of-the-art methods. Next, we analyze the comparison results and elaborate the ablation experiments in order to verify the effectiveness of the different modules in our framework. Finally, we compare the Inter-TRM with other graph neural networks to further prove the advantages of Inter-TRM.

### 4.1. Datasets

**PISC:** The PISC dataset contains a huge number of samples collected from various social media. It proposes a hierarchy task structure, namely PISC-C (three coarse-level relationships) and PISC-F (six fine-level relationships). In detail, coarse-level relationships are made up of an intimate relationship, non-intimate relationship and no relationship, while fine-level relationships consist of friend, family, couple, professional, commercial and no relation. Referring to the mainstream SRR methods [12,17,18,19,29], we adopt the mean average precision (mAP) as the evaluation metric.

**PIPA:** Zhang et al. [44] annotated bounding boxes of persons from Flickr photo albums and Sun et al. [21] further extended them as new SRR datasets, i.e., PIPA datasets. According to the social domain theory [45], PIPA divides social relationships into five social domains and further defines a subclassification with 16 specific social relationships. Referring to the mainstream SRR methods [12,17,18,19,29], we evaluate the proposed model only for 16 social relations, which employ top-1 accuracy (Acc) as the evaluation metric.

Other details of these two datasets, including the split of the training set, validation set and testing set, are shown in Table 2. For the PISC-C task, the training set consists of 13,142 images with 49,017 relation samples. The validation set and testing set include 4000 images with 14,536 relation samples and 15,497 relation samples, respectively. For the PISC-F task, the dataset can be divided into a training set of 16,828 images with 55,400 relation samples, a validation set of 500 images with 1505 relation samples and a testing set of 1250 images with 3961 relation samples. For the PIPA task, the training set, validation set and testing set are made up of 5857 images with 13,672 relation samples, 261 images with 709 relation samples and 2452 images with 5106 relation samples.

### 4.2. Implementation Details

In the training process, different components of our framework are trained simultaneously with the Adam [46] optimizer on one Nvidia GeForce RTX 2080 Ti GPU. The whole model is firstly trained with $lr=10^{-3}$ and then fine-tuned with $lr=10^{-4}$, while the *lr* reduces to one-tenth by 20 epochs. In addition, the learning attenuation, batch size and the maximum epoch are set as $5\times 10^{-4}$, 16 and 200, respectively.

In detail, we evaluate the model on the validation set after every epoch during the training process and pick the best model of the validation set within maximum epoch under $lr=10^{-3}$. Then, we fine-tune the chosen best model with $lr=10^{-4}$ and pick the best model of the validation set within maximum epoch again. Finally, we test the fine-tuned best model on the testing set and obtain the final experimental results.

At the same time, suggested by the collector of the PISC [12], data augmentation methods, such as pair-wise label reversing and the whole image horizontal rotation, are employed on those classes with fewer samples, e.g., the commercial relationship in the PISC-F task. The augmentation methods increase the number of samples in the tail classes and thus mitigate the imbalance of data to some extent.

### 4.3. Comparison Experiments with the State-of-the-Art Methods

To evaluate the effectiveness of our designed MT-SRR, we firstly brief the existing methods and then compare our final model with several state-of-the-art models, as shown in Table 3. The experimental results are presented against three different SRR tasks, namely PISC-C, PISC-F and PIPA. In detail, for the PISC-C task, Int., Non. and No. denote the three coarse-level relation classes ‘*intimate*’, ‘*non-intimate*’ and ‘*no relation*’, respectively. For the PISC-F task, Fri., Fam., Cou., Pro., Com. and No. are the six fine-level relation classes ‘*friend*’, ‘*family*’, ‘*couple*’, ‘*professional*’, ‘*commercial*’ and ‘*no relation*’, respectively. The quantities under these columns represent the per-class recall, while mAP is adopted as the whole evaluation metrics both for the PISC-C task and the PISC-F task. For the PIPA task, we adopt Acc to evaluate the whole framework as mentioned in Section 4.1. Finally, we further analyze the pros and cons of our proposed model on the fine-grained tasks in order to better understand the characteristics of the model.

1. **Dual-Glance** [12]. This method is the baseline method proposed by the collector of the PISC dataset. The attention mechanism is employed to fuse two kinds of features (named two glance), i.e., the features of persons and the features of contextual objects. Specifically, the features of persons including the feature of an individual, the feature extracted from union regions of two individuals and the coordinates of two individuals.
2. **DSFS** [16]: This method proposes a deep supervised feature selection framework, which learns a sparse weighting matrix to select the optimal feature subsets in order to reduce the noises and redundancy caused by high-dimension multi-source attributes.
3. **GRM** [29]. This method introduces stacked GGNNs to model the connections among person nodes and contextual objects nodes by a message-passing mechanism. Compared with the Dual-Glance, this method pays more attention to exploiting the interaction between the contextual objects and the persons.
4. **MGR** [25]. This method designs two different graph structures, namely the person–object graph and person–pose graph in order to exploit the connections between the person and the object as well as to utilize pose information, respectively. Two GCNs are then employed to exploit the topology information hidden in a graph structure and the outputs of the two GCNs are fused with the scene feature extracted from the whole image.
5. **SRG-GN** [15]. This method extends the traditional framework to a multi-task framework, which introduces five CNN-based extraction networks for person-pair attributions (i.e., age, gender and clothing) and relation attributions (i.e., scene and activity). Gated Recurrent Units (GRUs) are then adopted to fuse these different attributes and a multi-task loss is designed for relation classification.
6. **GR2N** [19]. This method constructs several virtual relation graphs in order to grasp the logical constraints among various relationships in the same image. GNNs are adopted to model the edges in the graph, using relations as nodes, which represent the logical constraints among the relations.
7. **SRR-LGR** [17]. This method further analyzes two different graph networks, i.e., a GCN and GGNN, and draws a conclusion that a GCN exploits the global features of the entire graph, while a GGNN focuses on the local message passing. A new reasoning module with the fusion of a GCN and GGNN is then proposed for SRR, dubbed local–global information reasoning.
8. **HF-SRGR** [18]. This method takes the different influences a scene exerts on different relationships in an image into consideration. On this basis, a variant of a GGNN is proposed, which introduces the attention mechanism to attentionally pass messages between person nodes and the scene node.

As shown in Table 3, our proposed MT-SRR significantly outperforms the state-of-the-art methods on the benchmark datasets for SRR. To be specific, the final model achieves 86.8%, 74.6% and 72.1% for the PISC-C task, PISC-F task and PIPA task, which exceeds the state-of-the-art methods by 2.0%, 1.3% and 6.4%, respectively. Note that MT-SRR achieves great improvement without introducing new types of features, and attributes such as age and gender are not used for a better comparison with the state-of-the-art methods [17,18], which further prove the effectiveness of the whole proposed model.

**Figure 6:**
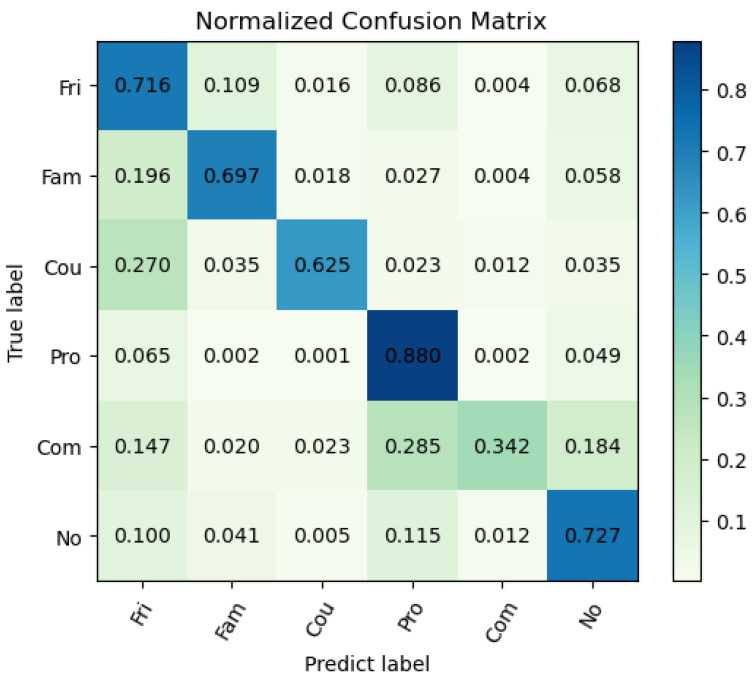
Confusion matrix of PISC-Fine task. The values on the leading diagonal denote the per-class recall.

**Figure 7:**
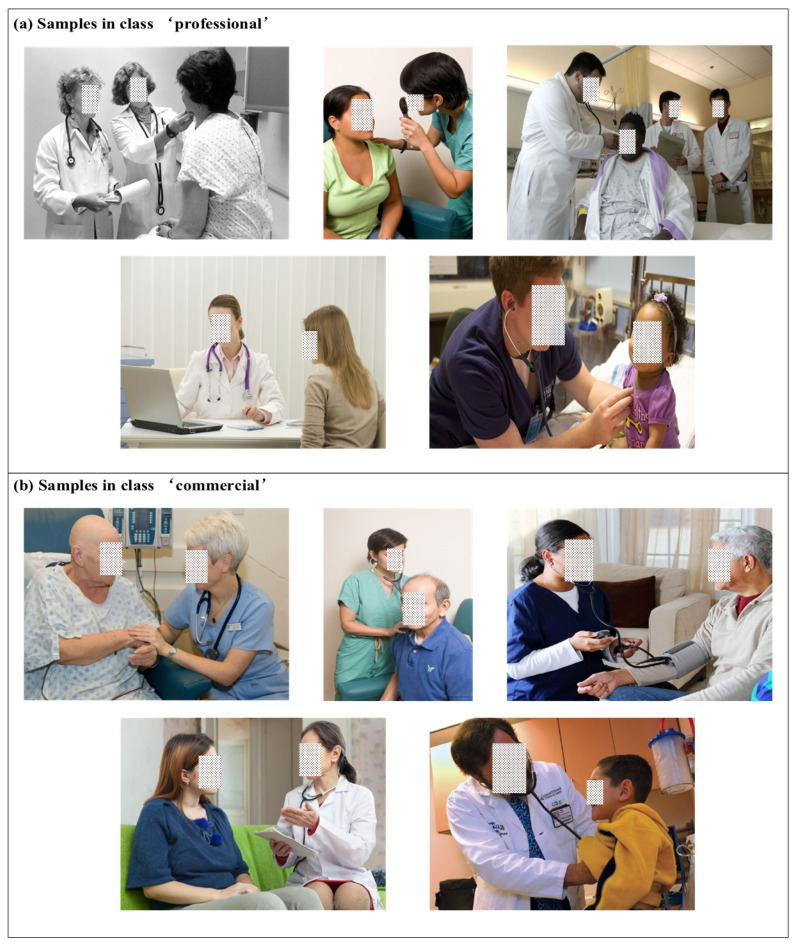
Similar samples in different classes.

In order to further analyze the characteristics of the proposed model, we consider the per-class recall in the PISC-F task, which is a fine-grained classification task. As shown in Table 3, our model achieves relatively better per-class recall, especially in the class ‘*professional*’ and the class ‘*no relation*’. However, the recall of class ‘*commercial*’ is relatively low, which means our model has trouble recognizing the samples of commercial relationships. To find out, we construct the confusion matrix, as shown in Figure 6. It can be easily observed that the confusion of the class ‘*commercial*’ mainly appears in the class ‘*professional*’. After the analysis, we partially owe the problem to the highly similar visual clues of these two classes and the overwhelming gap in the sample number of the class ‘*professional*’ over the class ‘*commercial*’ (the problem of imbalanced data will be further analyzed in Section 4). As shown in Figure 7, the samples annotated with the class ‘*professional*’ and the class ‘*commercial*’ both describe the relationships between patients and doctors, which have highly similar visual clues and thus make it difficult for our model to distinguish them. Although we have changed the loss function to mitigate the problem, highly similar visual clues and the imbalanced data still lead to the confusion between the ‘*commercial*’ and the ‘*professional*’ classes.

**Figure 8:**
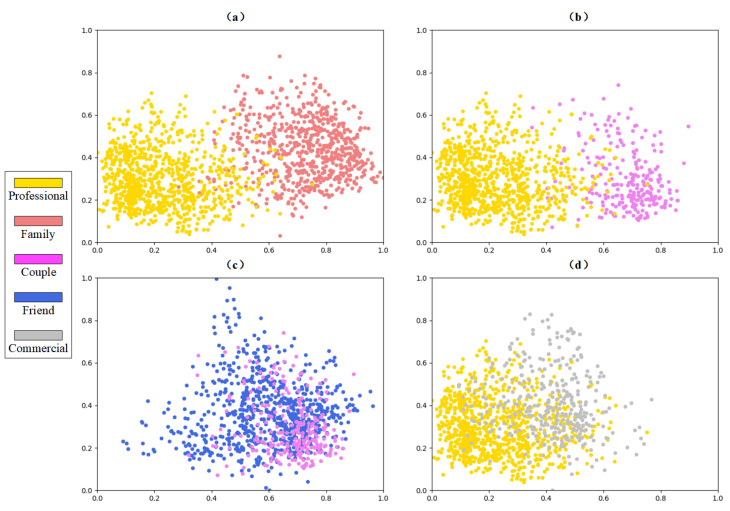
The distribution of social relation representations (outputs of Inter-TRM) in latent feature space. The points with different colors are the social relation representations from different classes. Note that, here, we utilize principal components analysis (PCA) to achieve dimensionality reduction. (**a**) Visualization of ‘*professional*’ and ‘*family*’. (**b**) Visualization of ‘*professional*’ and ‘*couple*’. (**c**) Visualization of ‘*friend*’ and ‘*couple*’. (**d**) Visualization of ‘*professional*’ and ‘*commercial*’.

To explain it more intuitively, we further analyze the outputs of Inter-TRM in the PISC-F task, namely the feature vectors that represent different social relationships. We first utilize a principal components analysis (PCA) to reduce the dimension of the features from 2048 to 2 and then scale the values to interval [0,1]. For better observation, we simultaneously exhibit two pairs of distinguishable classes (‘*professional*’ and ‘*family*’, ‘*professional*’ and ‘*couple*’) and two pairs of highly confused classes (‘*friend*’ and ‘*couple*’, ‘*professional*’ and ‘*commercial*’), as shown in Figure 8. Obviously, two classes with lower confusion (pairs of classes in Figure 8a,b) have less overlaps than those highly confused classes (pairs of classes in Figure 8c,d), which proves that the high similarities among indistinguishable classes deteriorate the recognition performance and lead to high inter-class confusion.

### 4.4. Ablation Study

In this subsection, we implement extensive experiments to evaluate the effectiveness of the different components in our framework. The detailed settings of the ablation experiments by removing different modules are as follows:

1. **Feature extraction using ViT (FE-ViT).** We simply concatenate four intra-relation features extracted by ViT and one scene feature extracted by ResNet-50 for relation classification.
2. **Feature extraction + Intra-TRM (FE-ViT + Intra-TRM).** We added Inter-TRM on top of the ablation (i), which dynamically fuses four intra-relation features and one scene feature.
3. **Feature extraction + Intra-TRM + Inter-TRM (FE-ViT + Intra-TRM + Inter-TRM).** We added Inter-TRM on the basis of ablation (ii). The outputs of Intra-TRM, namely the social relation representations in the same image, are fed to the Inter-TRM module for attentionally enhancing social relation representations of similar relationships.
4. **Feature extraction + Intra-TRM + Inter-TRM + loss with margin (FE-ViT + Intra-TRM + Inter-TRM + loss-m).** On top of ablation (iii), we replace the standard CE loss with the new loss with the margin. The details are in Section 3.4.

Table 4 lists the ablation results. We start with the baseline experiment ablation (i), which achieves 76.2%, 67.0% and 66.8% for PISC-C, PISC-F and PIPA, respectively. Compared with ablation (i), ablation (ii) achieves improvement with absolute 5.9%, 5.4% and 2.5% for PISC-C, PISC-F and PIPA, which demonstrates that Intra-TRM can effectively fuse intra-relation features and scene features. Ablation (iii) attentionally enhances the representation of similar relationships in the same image. The effects are boosted up to 85.7%, 75.1% and 71.4%. The change of the CE to the new loss with margin in ablation (iv) promotes the results further on PISC-C and PIPA by 1.1% and 0.7%, respectively. However, for the PISC-F task, the overall mAP decreases from 75.1% to 74.6%. This will be further analyzed below.

To better understand the influence of the new loss function, we further compare ablation (iii) and ablation (iv) on the PISC-F dataset, as shown in Table 5. With the new loss function, MT-SRR increases the recall of most classes, except for the class ‘*no relation*’, of which the recalls of the minority classes ‘*couple*’ and ‘*commercial*’ increase by 19.5% and 2.8%, respectively. The overall accuracy of ablation (iv) is also 2.1% higher than ablation (iii), which demonstrates that the new loss can boost the performance of the whole model so as to make more correct predictions. However, for the minority class ‘*commercial*’, the increase in the recall is still not as large as expected. This is owing to the data augmentation strategy, which multiplies the sample number of the class ‘*commercial*’, as shown in Table 6. After the data augmentation, the sample number of the class ‘*commercial*’ increases from 523 to 8372, which decreases the margin (in Equation (7)) between the class ‘*commercial*’ and the highly similar class ‘professional’ in the new loss function. A lower margin between two classes weakens the capacity of our model to distinguish the class ‘*commercial*’ and ‘*professional*’ and thus leads to a lower increase in recall.

### 4.5. Comparison Experiments with Inter-TRM and Other Graph-Based Networks

In this subsection, we further compare our proposed Inter-TRM with other graph-based networks used in SRR in order to demonstrate that Inter-TRM can enhance the social relation representations by attentionally aggregating the representations of similar relationships in the same image; namely, it better exploits the logical constraints among relationships. To be specific, we compare our proposed Inter-TRM with a GGNN [47], GCN [48] and Graph Attention Network (GAT) [49] on the PISC-F task, as shown in Table 7. Among those graph-based networks, the GAT is the closest to our design idea of Inter-TRM because it introduces the attention mechanism into a graph structure. However, the nodes in the GAT only aggregate the information of the neighbor nodes, which limits the ability of global information aggregation. In [18], the GAT and GCN are also proved to be less efficient than the GGNN for constructed social relation graph reasoning because they are constructed based on spectral graph theory and thus pay more attention to the topological information. The results in Table 7 further demonstrate that our Inter-TRM module performs better than the GGNN, GCN and GAT in exploiting the logical constraints among relationships than the graph-based networks.

## 5. Conclusions

In this paper, we focus on the design of the feature fusion module, which orchestrates the intra-relation features, inter-relation features and scene feature in order to generate more rational social relation representation for a deeper understanding of SRR. Specially, two transformer-based feature fusion modules, namely Intra-TRM and Inter-TRM, are introduced to dynamically fuse all the features for social relation representations generation and attentionally enhance the representations of similar social relationships in the same image, respectively. We also add a newly designed margin to standard CE in order to mitigate the bad effect caused by imbalanced data. The new margin can be potentially used in different tasks which have the same problem, e.g., emotion recognition and activity recognition in a public space.

In total, the two transformer-based modules boost the performance with absolute 9.5%, 8.1% and 4.6% for PISC-C (mAP), PISC-F (mAP) and PIPA (Acc) over the ablation baseline, which demonstrates that our MT-SRR can efficiently orchestrate the features on different scales. The comparison between Inter-TRM and graph-based networks further proves that Inter-TRM is the better choice for exploiting the logical constraints. In addition, the ablation results also prove that the newly designed margin can alleviate the bad effect caused by imbalanced data and improve the recognition accuracy on three tasks with only 0.5% deterioration on PISC-F (mAP). In general, our proposed MT-SRR significantly outperforms the state-of-the-art methods by absolute 2.0%, 1.3% and 6.4% for PISC-C (mAP), PISC-F (mAP) and PIPA (Acc), which illustrates the effectiveness of our proposed MT-SRR.

However, some classes with highly similar visual clues still suffer from low recognition accuracy. To address the problem, how to comprehensively utilize multimodal social data (text, audio, etc.) to distinguish the highly confused classes and achieve more accurate recognition is thus a key issue in the future. In addition, how to apply SRR to higher-level social scene understanding and further benefit more complex social intelligence systems, such as a city-scale public administration system, is another key issue for future research.

## Figures and Tables

**Table 1:** Features on different scales for SRR.

Features on Different Scales	Category
Intra-relation feature	Individual features: face [23,26,27,28]; gesture [25]; cropped individual region [12,15,17,25]
	Person-pair features: cropped union region [12,15,17,18,25]; relative position [12,17,18,25]
	Attributes: age [15,16]; gender [15,16]
Inter-relation feature	Logical constraint [17,18,19]
Scene feature	Contextual object [12]
	Whole scene (whole image) [18,19,25]

**Table 2:** The split details according to different social relation tasks.

	Training		Validation		Testing	
	Images	Relations	Images	Relations	Images	Relations
PISC-C	13,142	49,017	4000	14,536	4000	15,497
PISC-F	16,828	55,400	500	1505	1250	3961
PIPA	5857	13,672	261	709	2452	5106

**Table 3:** The comparison results (per-class recall (in %) and the mAP over all classes (in %) for PISC and Acc (in %) for PIPA) of our MT-SRR model with the state-of-the-art methods on PISC-C, PISC-F and PIPA datasets. Best results are highlighted in bold face. (Int.: ‘*intimate*’, Non.: ‘*non-intimate*’, No.: ‘*no relation*’, Fri.: ‘*friend*’, Fam.: ‘*family*’, Cou.: ‘*couple*’, Pro.: ‘professional’, Com.: ‘*commercial*’).

	PISC-C				PISC-F							PIPA
	Int.	Non.	No.	mAP	Fri.	Fam.	Cou.	Pro.	Com.	No.	mAP	Acc
Dual-Glance [12]	73.1	84.2	59.6	79.7	35.4	68.1	76.3	70.3	57.6	60.9	63.2	59.6
DSFS [16]	-	-	-	-	-	-	-	-	-	-	-	61.5
GRM [29]	81.7	73.4	65.5	82.8	59.6	64.4	58.6	76.6	39.5	67.7	68.9	62.3
MGR [25]	-	-	-	-	64.6	67.8	60.5	76.8	34.7	70.4	70.0	64.4
SRG-GN [15]	-	-	-	-	25.2	80.0	100.0	78.4	83.3	62.5	71.6	53.6
GR2N [19]	81.6	74.3	70.8	83.1	60.8	65.9	84.8	73.0	51.7	70.4	72.7	64.3
SRR-LGR [17]	89.6	84.6	78.5	84.8	83.9	52.4	35.9	64.0	54.0	63.6	73.0	66.1
HF-SRGR [18]	89.1	87.0	75.5	84.6	82.2	39.4	33.2	60.0	47.7	71.8	73.3	65.9
Ours	91.8	91.8	75.2	**86.8**	71.6	69.7	62.5	88.0	34.2	72.7	**74.6**	**72.5**

**Table 4:** Ablation results with row 1 to row 5 corresponding to the results of ablation (i) to ablation (v). The best results are given in bold.

Ablation Methods	PISC-C (mAP)	PISC-F (mAP)	PIPA (Acc)
(i) FE-ViT	76.2	67.0	66.8
(ii) FE-ViT + Intra-TRM	82.1	72.4	69.3
(iii) FE-ViT + Intra-TRM + Inter-TRM	85.7	**75.1**	71.4
(iv) FE-ViT + Intra-TRM + Inter-TRM + loss-m	**86.8**	74.6	**72.1**

**Table 5:** Detailed comparison between ablation (iii) and ablation (iv) on PISC-F dataset. Ablation (iii) consists of FE-ViT, Intra-TRM and Inter-TRM, while ablation (iv) introduces the new loss function into the training process on basis of ablation (iii). Better results are given in bold.

Ablation Methods	Fri.	Fam.	Cou.	Pro.	Com.	No.	mAP	Acc
Ablation(iii)	70.5	68.1	43.0	80.4	31.4	**78.3**	**75.1**	69.0
Ablation(iv)	**71.6**	**69.7**	**62.5**	**88.0**	**34.2**	72.7	74.6	**71.2**

**Table 6:** The sample distribution of the PISC dataset. Row 1 denotes the sample numbers of the original dataset, while row 2 shows the sample numbers after the data augmentation strategy is employed.

Social Relations	Fri.	Fam.	Cou.	Pro.	Com.	No.
Numbers of samples (Origin dataset)	12,686	7818	1552	20,842	523	11,979
Numbers of samples (After data augmentation)	13,120	7982	3149	21,448	8372	18,541

**Table 7:** Comparative results between Inter-TRM and other graph-based networks on PISC-F. The best results are given in bold.

Ablation Methods	PISC-F (mAP)
GCN	71.5
GAT	72.6
GGNN	73.1
Inter-TRM	**74.6**

## Data Availability

The People in Social Context (PISC) [12] dataset is available on the website https://zenodo.org/record/1059155 and the People in Photo Album (PIPA) dataset [21] is available on the website https://www.mpi-inf.mpg.de/social-relation.

## Abbreviations

The following alphabetical abbreviations are used in this manuscript:

Acc	Top-1 accuracy
CE	Cross-entropy
CNN	Convolutional neural network
Com.	Class ‘*commercial*’
Cou.	Class ‘*couple*’
Fam.	Class ‘*family*’
FC	Fully connected layer
Fri.	Class ‘*friend*’
GAT	Graph attention network
GCN	Graph convolutional network
GGNN	Gated graph neural network
GNN	Graph neural network
Int.	Class ‘*intimate*’
Inter-TRM	Inter-relation transformer
Intra-TRM	Intra-relation transformer
LN	Layer normalization
*lr*	Learning rate
mAP	Mean average precision
MLP	Multi-layer perception
MSA	Multi-head self-attention
MT-SRR	Multi-level transformer-based social relation recognition
NLP	Natural language processing
No.	Class ‘*no relation*’
Non.	Class ‘*non-intimate*’
PIPA	People in Photo Album
PISC	People in Social Context
PISC-C	Coarse-level social relation recognition task in People in Public Space dataset
PISC-F	Fine-level social relation recognition task in People in Public Space dataset
Pro.	Class ‘*professional*’
SRR	Social relation recognition
ViT	Vision transformer

## References

[1] Umberson D., Montez J.K. (2010). Social Relationships and Health: A Flashpoint for Health Policy. J. Health Soc. Behav..

[2] Ramanathan V., Yao B., Li F.F. Social Role Discovery in Human Events. Proceedings of the 2013 IEEE Conference on Computer Vision and Pattern Recognition (CVPR).

[3] Quiroz M., Patiño R., Diaz-Amado J., Cardinale Y. (2022). Group Emotion Detection Based on Social Robot Perception. Sensors.

[4] Sou K., Shiokawa H., Yoh K., Doi K. (2021). Street Design for Hedonistic Sustainability through AI and Human Co-Operative Evaluation. Sustainability.

[5] Rato D., Prada R. (2021). Towards Social Identity in Socio-Cognitive Agents. Sustainability.

[6] Hou Q., Han M., Cai Z. (2020). Survey on data analysis in social media: A practical application aspect. Big Data Min. Anal..

[7] Li W., Zlatanova S. (2021). Significant Geo-Social Group Discovery over Location-Based Social Network. Sensors.

[8] Minetto A., Nardin A., Dovis F. (2021). Modelling and Experimental Assessment of Inter-Personal Distancing Based on Shared GNSS Observables. Sensors.

[9] Liu M., Quan Z.W., Wu J.M., Liu Y., Han M. (2022). Embedding temporal networks inductively via mining neighborhood and community influences. Appl. Intell..

[10] Guo X., Xiang Y., Chen Q. A vector space model approach to social relation extraction from text corpus. Proceedings of the 2011 Eighth International Conference on Fuzzy Systems and Knowledge Discovery (FSKD).

[11] Cernian A., Vasile N., Sacala I.S. (2021). Fostering Cyber-Physical Social Systems through an Ontological Approach to Personality Classification Based on Social Media Posts. Sensors.

[12] Li J., Wong Y., Zhao Q., Kankanhalli M. Dual-Glance Model for Deciphering Social Relationships. Proceedings of the 2017 IEEE International Conference on Computer Vision (ICCV).

[13] Dai P., Lv J., Wu B. Two-Stage Model for Social Relationship Understanding from Videos. Proceedings of the 2019 IEEE International Conference on Multimedia and Expo (ICME).

[14] Qing L., Li L., Xu S., Huang Y., Liu M., Jin R., Liu B., Niu T., Wen H., Wang Y. Public Life in Public Space (PLPS): A multi-task, multi-group video dataset for public life research. Proceedings of the IEEE/CVF International Conference on Computer Vision (ICCV) Workshops.

[15] Goel A., Ma K.T., Tan C. An End-To-End Network for Generating Social Relationship Graphs. Proceedings of the 2019 IEEE/CVF Conference on Computer Vision and Pattern Recognition (CVPR).

[16] Wang M., Du X., Shu X., Wang X., Tang J. (2020). Deep supervised feature selection for social relationship recognition. Pattern Recognit. Lett..

[17] Qing L., Li L., Wang Y., Cheng Y., Peng Y. (2021). SRR-LGR: Local–Global Information-Reasoned Social Relation Recognition for Human-Oriented Observation. Remote Sens..

[18] Li L., Qing L., Wang Y., Su J., Cheng Y., Peng Y. (2021). HF-SRGR: A new hybrid feature-driven social relation graph reasoning model. Vis. Comput..

[19] Li W., Duan Y., Lu J., Feng J., Zhou J. Graph-based social relation reasoning. Proceedings of the 16th European Conference on Computer Vision (ECCV).

[20] Dosovitskiy A., Beyer L., Kolesnikov A., Weissenborn D., Zhai X., Unterthiner T., Dehghani M., Minderer M., Heigold G., Gelly S. An Image is Worth 16x16 Words: Transformers for Image Recognition at Scale. Proceedings of the Ninth International Conference on Learning Representations (lCLR).

[21] Sun Q., Schiele B., Fritz M. A Domain Based Approach to Social Relation Recognition. Proceedings of the 2017 IEEE Conference on Computer Vision and Pattern Recognition (CVPR).

[22] Fang R., Tang K.D., Snavely N., Chen T. Towards computational models of kinship verification. Proceedings of the 2010 IEEE International Conference on Image Processing (ICIP).

[23] Dibeklioglu H., Salah A.A., Gevers T. Like father, like son: Facial expression dynamics for kinship verification. Proceedings of the 2013 IEEE International Conference on Computer Vision (ICCV).

[24] Gao J., Qing L., Li L., Cheng Y., Peng Y. (2021). Multi-scale features based interpersonal relation recognition using higher-order graph neural network. Neurocomputing.

[25] Zhang M., Liu X., Liu W., Zhou A., Ma H., Mei T. Multi-Granularity Reasoning for Social Relation Recognition From Images. Proceedings of the 2019 IEEE International Conference on Multimedia and Expo (ICME).

[26] Wang G., Gallagher A., Luo J., Forsyth D. Seeing people in social context: Recognizing people and social relationships. Proceedings of the 11th European Conference on Computer Vision (ECCV).

[27] Xia S., Shao M., Luo J., Fu Y. (2012). Understanding kin relationships in a photo. IEEE Trans. Multimed..

[28] Lu J., Zhou X., Tan Y., Shang Y., Zhou J. (2014). Neighborhood Repulsed Metric Learning for Kinship Verification. IEEE Trans. Pattern Anal. Mach. Intell..

[29] Wang Z., Chen T., Ren J., Yu W., Cheng H., Lin L. Deep reasoning with knowledge graph for social relationship understanding. Proceedings of the 27th International Joint Conference on Artificial Intelligence (IJCAI).

[30] Wu H., Codella N., Liu M., Dai X., Yuan L., Zhang L. CvT: Introducing Convolutions to Vision Transformers. Proceedings of the 2021 IEEE/CVF International Conference on Computer Vision (ICCV).

[31] Liu Z., Lin Y., Cao Y., Hu H., Wei Y., Zhang Z., Lin S., Guo B. Swin Transformer: Hierarchical Vision Transformer using Shifted Windows. Proceedings of the 2021 IEEE/CVF International Conference on Computer Vision (ICCV).

[32] Wang W., Xie E., Li X., Fan D.P., Song K., Liang D., Lu T., Luo P., Shao L. Pyramid Vision Transformer: A Versatile Backbone for Dense Prediction without Convolutions. Proceedings of the 2021 IEEE/CVF International Conference on Computer Vision (ICCV).

[33] Wang L., Li R., Wang D., Duan C., Wang T., Meng X. (2021). Transformer Meets Convolution: A Bilateral Awareness Network for Semantic Segmentation of Very Fine Resolution Urban Scene Images. Remote Sens..

[34] Bazi Y., Bashmal L., Rahhal M.M.A., Dayil R.A., Ajlan N.A. (2021). Vision Transformers for Remote Sensing Image Classification. Remote Sens..

[35] Zhang J., Zhao H., Li J. (2021). TRS: Transformers for Remote Sensing Scene Classification. Remote Sens..

[36] He X., Zhou Y., Zhao J., Zhang D., Yao R., Xue Y. (2022). Swin Transformer Embedding UNet for Remote Sensing Image Semantic Segmentation. IEEE Trans. Geosci. Remote Sens..

[37] Qiu H., Hou B., Ren B., Zhang X. (2022). Spatio-Temporal Tuples Transformer for Skeleton-Based Action Recognition. arXiv.

[38] Li X., Hou Y., Wang P., Gao Z., Xu M., Li W. (2022). Trear: Transformer-Based RGB-D Egocentric Action Recognition. IEEE Trans. Cogn. Dev. Syst..

[39] Bai R., Li M., Meng B., Li F., Ren J., Jiang M., Sun D. (2022). GCsT: Graph Convolutional Skeleton Transformer for Action Recognition. arXiv.

[40] Zhou B., Lapedriza A., Khosla A., Oliva A., Torralba A. (2018). Places: A 10 million image database for scene recognition. IEEE Trans. Pattern Anal. Mach. Intell..

[41] Deng J., Dong W., Socher R., Li L., Li K., Li F. ImageNet: A large-scale hierarchical image database. Proceedings of the 2009 IEEE Conference on Computer Vision and Pattern Recognition (CVPR).

[42] Devlin J., Chang M.W., Lee K., Toutanova K. BERT: Pre-training of Deep Bidirectional Transformers for Language Understanding. Proceedings of the 2019 Conference of the North American Chapter of the Association for Computational Linguistics: Human Language Technologies.

[43] Feng C., Zhong Y., Huang W. Exploring Classification Equilibrium in Long-Tailed Object Detection. Proceedings of the 2021 IEEE/CVF International Conference on Computer Vision (ICCV).

[44] Zhang N., Paluri M., Taigman Y., Fergus R., Bourdev L. Beyond frontal faces: Improving person recognition using multiple cues. Proceedings of the 2015 IEEE Conference on Computer Vision and Pattern Recognition (CVPR).

[45] Bugental D.B. (2000). Acquisition of the algorithms of social life: A domain-based approach. Psychol. Bull..

[46] Kingma D., Ba J. Adam: A method for stochastic optimization. Proceedings of the 3rd International Conference on Learning Representations (ICLR).

[47] Li Y., Zemel R., Brockschmidt M., Tarlow D. Gated Graph Sequence Neural Networks. Proceedings of the 4th International Conference on Learning Representation (ICLR).

[48] Kipf T.N., Welling M. Semi-supervised classification with graph convolutional networks. Proceedings of the 5th International Conference on Learning Representation (ICLR).

[49] Veličković P., Preixens G.C., Paga A.C., Romero A., Liò P., Bengio Y. Graph attention networks. Proceedings of the International Conference on Learning Representations (ICLR).

